# By what molecular mechanisms do social determinants impact cardiometabolic risk?

**DOI:** 10.1042/CS20220304

**Published:** 2023-03-24

**Authors:** Yvonne Baumer, Mario A. Pita, Andrew S. Baez, Lola R. Ortiz-Whittingham, Manuel A. Cintron, Rebecca R. Rose, Veronica C. Gray, Foster Osei Baah, Tiffany M. Powell-Wiley

**Affiliations:** 1 Social Determinants of Obesity and Cardiovascular Risk Laboratory, Cardiovascular Branch, Division of Intramural Research, National Heart Lung and Blood Institute, National Institutes of Health, Bethesda, MD, U.S.A.; 2 Intramural Research Program, National Institute on Minority Health and Health Disparities, National Institutes of Health, Bethesda, MD, U.S.A.

**Keywords:** Cardiometabolic Disease, Chronic Stress, Immune System, Inflammation, Psychosocial and Environmental Stressors, Social Determinants of Health

## Abstract

While it is well known from numerous epidemiologic investigations that social determinants (socioeconomic, environmental, and psychosocial factors exposed to over the life-course) can dramatically impact cardiovascular health, the molecular mechanisms by which social determinants lead to poor cardiometabolic outcomes are not well understood. This review comprehensively summarizes a variety of current topics surrounding the biological effects of adverse social determinants (i.e., the biology of adversity), linking translational and laboratory studies with epidemiologic findings. With a strong focus on the biological effects of chronic stress, we highlight an array of studies on molecular and immunological signaling in the context of social determinants of health (SDoH). The main topics covered include biomarkers of sympathetic nervous system and hypothalamic–pituitary–adrenal axis activation, and the role of inflammation in the biology of adversity focusing on glucocorticoid resistance and key inflammatory cytokines linked to psychosocial and environmental stressors (PSES). We then further discuss the effect of SDoH on immune cell distribution and characterization by subset, receptor expression, and function. Lastly, we describe epigenetic regulation of the chronic stress response and effects of SDoH on telomere length and aging. Ultimately, we highlight critical knowledge gaps for future research as we strive to develop more targeted interventions that account for SDoH to improve cardiometabolic health for at-risk, vulnerable populations.

## Introduction

The social determinants of health (SDoH) include several psychosocial and environmental factors that influence health and functioning of individuals and communities. A SDoH framework described by Powell-Wiley et al. [1] incorporates structural determinants such as socioeconomic status (SES) and neighborhood environment, and intermediary determinants, including the social environment, and lived personal experiences of discrimination (Supplementary Figure S1). SDoH can serve as sources of chronic psychosocial and environmental stress (Supplementary Figure S1) and are associated with a variety of biological sequelae; the harmful biological effects of SDoH are known as the biology of adversity [2]. Specifically, poor social conditions are associated with disparities in cardiovascular disease (CVD) outcomes (Supplementary Figure S1) [1,3]. Furthermore, the COVID-19 pandemic has highlighted the importance of addressing the biologic consequences of social adversities that perpetuate health disparities, given that lower-resourced populations and those most impacted by systemic inequalities were at higher risk for severe COVID-19 and death [4]. Moreover, the COVID-19 pandemic and its bidirectional interactions with cardiometabolic diseases and socioeconomic factors call attention to the interplay between biologic, social, economic, and environmental factors that influence health [5].

In this review, we explore the biomolecular mechanisms underlying the health effects of SDoH, with a focus on CVD, and highlight research on biomarkers and signaling pathways of the stress response. Chronic stress and its effects on the body have been found to be important factors in the mechanisms by which SDoH connect to CVD [6,7]. A comprehensive overview of the cellular signaling that links known associations between SDoH, chronic stress and adversity, and CVD as a key health outcome is currently lacking. Thus, we outline what is currently known about these associations and signaling pathways downstream of psychosocial and environmental stressors (PSES) as SDoH to provide a foundation guiding future research toward addressing these important gaps in our knowledge. A greater understanding of these signaling mechanisms may improve the ways in which we understand SDoH clinically, potentially steering us towards more useful biomarkers of SDoH to better inform patient care or aid in the development of targeted interventions. In this review, we focus on signaling derived from chronic activation of the stress response, and discuss how PSES as SDoH relate to inflammation, cellular function, methylation, telomere length, and biological aging.

## Biomarkers of stress and the biology of adversity

**Figure 1:**
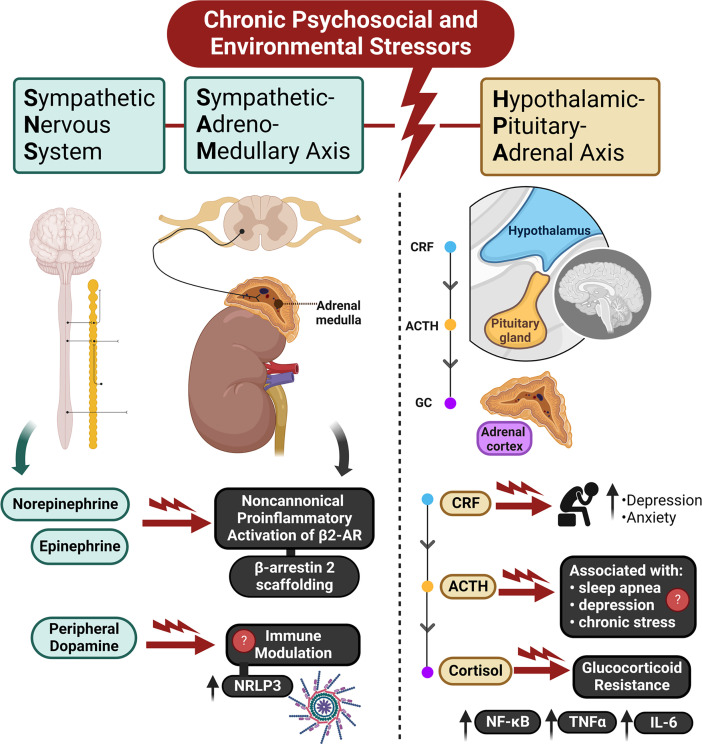
Effects of chronic psychosocial and environmental stressors (PSES) on the stress response From left to right: Activation of the SNS releases catecholamines, mainly norepinephrine and epinephrine, from postganglionic sympathetic nerve terminals which act on adrenergic receptors. Activation of the sympathetic-adrenomedullary (SAM) axis releases catecholamines into circulation via the adrenal medulla. Chronic activation of these stress responses and repeated release of catecholamines may lead to a noncanonical pro-inflammatory response facilitated by β2-adrenergic receptors. Release of dopamine from sympathetic nerves may also play a role in the effects of chronic stress, particularly via immune modulation and increased activity of the NRLP3 inflammasome. The HPA axis is also activated by stress and results in the release of CRF, ACTH, and cortisol. Each hormone has been associated with adverse effects of chronic stress. Most notably, glucocorticoid resistance leads to an increase in pro-inflammatory signaling, with increased levels of NF-κB, TNFα, and IL-6.

As the human body encounters PSES, the stress response includes coordinated activation of both the hypothalamic–pituitary–adrenal (HPA) axis and the sympathetic nervous system (SNS), including the sympatho-adrenomedullary (SAM) axis [8,9] as depicted in Figure 1. In this section, we highlight the signaling pathways initiated by known biomarkers of stress. Due to their connection to the stress response, these signaling pathways may help us understand how stressors stemming from SDoH may contribute to the biology of adversity.

### Biomarkers of sympathetic nervous system activation

The SNS response is mediated by the noradrenergic system [9]. Neural projections travel from the paraventricular nucleus of the hypothalamus, the locus coeruleus, and the rostral ventrolateral medulla to pre-ganglionic sympathetic neurons located in the dorsal intermediolateral cellular column of the spinal cord [10]. Each of these pre-ganglionic fibers then connect to several post-ganglionic neurons in one or many pre-spinal ganglia or sympathetic paravertebral nuclei [10]. Other pre-ganglionic neurons synapse directly with chromaffin cells in the adrenal medulla, which synthesize and secrete the catecholamines epinephrine (Epi) and norepinephrine (NE) [10]. Catecholamines, mainly characterized in the setting of the ‘fight or flight’ response [11], can function as hormones or as neurotransmitters. In the context of the biology of adversity, studies have shown that PSES associate with increased urinary catecholamines [12–15]. The effects of chronic stress on the SNS and cellular adaptations that occur afterwards have also been investigated [16], and specifically in relation to CVD [17].

#### Norepinephrine and epinephrine

NE and Epi signal through a class of G-protein-coupled receptors called adrenergic receptors (ARs). ARs are divided into α (α1/2-AR) and β (β1-3-AR) ARs. NE is known to primarily stimulate α1/2-ARs well as β1-AR. In contrast, Epi can stimulate all subtypes of α and β-ARs but with a stronger binding capacity to β2/3-ARs [18–20]. β2-AR signaling increases intracellular cAMP levels, activating PKA and subsequently phosphorylating CREB, a transcription factor. It has been well documented that immune cells express receptors for catecholamines and that the stress response may therefore have immunomodulatory effects [20]. Under resting conditions, cAMP-mediated signaling can suppress pro-inflammatory nuclear factor NF-κB (NF-κB) [21] in the classical activation of β-AR signaling. Interestingly, exposure to chronic PSES induces a signaling switch to noncanonical activation facilitated by β-arrestin 2 binding to β2-AR under high NE concentrations. This noncanonical activation alters signaling away from cAMP to ERK1/2 and MAPK signaling which has been reported to have a pro-inflammatory effect [22]. Additionally, a reduction in β-AR density on immune cells as well as a reduction in receptor sensitivity have been reported in the setting of chronic PSES [23,24].

#### Dopamine

Activation of the SNS has been shown to induce the release of dopamine (DA) from sympathetic nerve terminals, where DA may act peripherally on blood vessels and organs [25]. NE and Epi predominate in the stress response, though the role of peripheral DA in stress is still not completely understood. Plasma levels of DA, unlike levels of other stress hormones, appear to remain unchanged in the setting of insulin-induced hypoglycemia or acute mental stress, but increases in DA plasma levels were observed in humans after exercise or surgery [26]. Childhood sexual abuse as a form of chronic stress was associated with elevated levels of dopamine metabolites [27]. Like NE and Epi, DA receptors are expressed on immune cells and DA has been shown to have immunomodulatory effects, contributing to functional changes in T cells, B cells, macrophages, natural killer (NK) cells, and dendritic cells [28].

There are five known DA receptors (D1-5). These five G protein-coupled receptors are categorized into two classes, D1-like receptors (D1 and D5) and D2-like receptors (D2, D3, and D4). They are primarily distinguished by D1-like receptor activation leading to increased levels of cAMP and D2-like receptor activation inhibiting cAMP formation [29]. There are also distinct immunomodulatory effects of D1-like and D2-like receptor activation on numerous cell types, with many studies reporting anti-inflammatory effects [28]. However, additional studies are needed to examine the role of peripheral DA immunomodulation in the context of chronic stress, especially examining whether a noncanonical pro-inflammatory response is elicited similar to that of NE and β2-AR after repeated activation.

The effects of chronic stress on the dopaminergic system have been studied in the CNS, particularly in the context of psychosocial adversity and how it may lead to the development of mental illness [30]. A small case–control study using 3,4-dihydroxy-6-[18F]-fluoro-l-phenylalanine ([18F]-DOPA) positron emission tomography (PET) compared dopamine synthesis in the brain between low and high psychosocial adversity matched participants upon exposure to an acute stressor in the laboratory. While dopamine synthesis increased after the acute stressor among the low exposure group, those with long-term exposure to psychosocial adversity exhibited altered dopamine synthesis capacity with blunted subcortical dopamine transmission [30]. Such alterations to the dopaminergic system may lead to greater vulnerability to mental illness, though more investigation is needed. This vulnerability to mental illness is relevant in the context of SDoH. For instance, a large epidemiologic study using a nationally representative sample has demonstrated the intersectional effect of sociodemographic indicators of SDoH (race and ethnicity, and gender) when examining the risk of a major depressive episode, an important risk factor for CVD [31,32].

Köhler studied the relationship between early life adversity and dopamine 1-like receptor (D1LR) expression. He noted that long-term separation stress (LTSS) induced depressive-like behaviors, while short-term separation stress (STSS) reduced depressive-like behaviors in a mouse model. It was discovered that D1LR gene expression was increased, while 32-kDA dopamine and cAMP-regulated phosphoprotein (DARPP-32)-a modulator of D1LR activity-exhibited decreased gene expression in response to STSS. On the other hand, LTSS resulted in an increase in DARPP-32 gene expression without associated changes in D1LR gene expression. In the future, this relationship will have to be studied in greater breadth to help identify critical intervention periods [33]. Overall, examining the role of DA in psychosocial stress, both in the periphery and CNS, may unlock a greater understanding of multiple facets of the biology of adversity, from immunomodulation and inflammation to susceptibility to mental illness and CVD.

### Biomarkers of HPA axis activation

The HPA axis is initiated after the body encounters a stimulus that activates a stress state in the brain. Neuronal activity cumulates at the hypothalamus in order to elicit an endocrine response [34]. Corticotrophin-releasing factor (CRF) is secreted by the median paraventricular nucleus (PVN) of the hypothalamus into the vasculature and upon binding to CRF1 receptors (CRF1R) on the anterior pituitary, stimulates production of pro-opiomelanocortin (POMC) and adrenocorticotropic hormone (ACTH). ACTH then acts on the adrenal cortex to stimulate synthesis and secretion of glucocorticoids, namely cortisol in humans [35–38]. Glucocorticoids have various and wide-reaching effects on many cell types across the body but also play a crucial role in providing negative feedback to the HPA axis to tone down the stress response once activated. Chronic activation of the HPA axis may interfere with this negative feedback system, resulting in hypercortisolemia [39]. Long term HPA axis dysregulation disrupts homeostasis, contributing to physiological and mental/behavioral pathology [40]. One mechanism proposed for the development of pathology after chronic activation focuses on the effects of chronic stressors on the glucocorticoid receptor, causing glucocorticoid resistance (GCR) that results in dysregulation of the HPA axis and inflammation [41]. Additionally, when considering the effects of chronic stress, we must consider the interaction or cumulative effects of the HPA axis alongside the catecholaminergic systems, particularly when examining inflammation and immune homeostasis [42,43]. Next, we must also consider the unique sources of chronic stress that may act as SDoH and examine the signaling pathways which may relate to CVD and other associated pathologies.

While the associations between the HPA axis and CVD are becoming increasingly evident, the precise biological signaling mechanisms of these effects are not completely understood. Next in our review, signaling associated with key components of the HPA axis is explored in the context of SDoH and the biology of adversity, with a focus on cortisol, corticotropin-releasing factor (CRF), and adrenocorticotropic hormone (ACTH).

#### Cortisol

Cortisol, a glucocorticoid transported in the circulation by the corticosteroid-binding globulin (CBG), signals through the glucocorticoid receptor (GR) which, in the absence of glucocorticoids, is primarily located in a multi-protein complex within the cytoplasm. This multi-protein complex includes chaperone proteins (e.g., hsp90) as well as FK506 family proteins (e.g., FKBP51). Upon entry into the cytosol and binding to the GR, a conformational change initiates its translocation to the nucleus where it takes part in gene activation or repression by binding to glucocorticoid response elements (GREs); regulated genes include β-arrestin 2, osteocalcin, mitogen-activated protein kinase phosphatase-1 (MKP-1), or serum/glucocorticoid regulated kinase (SGK1). Glucocorticoid-regulated genes can mediate anti-inflammatory and anti-angiogenic effects as well as other important physiological processes like glucose homeostasis, lipid homeostasis, and cell survival [44–46]. Interestingly, the sensitivity of GRs can decrease after undergoing a hyperacetylation process resulting in GCR, which can lead to overactivation of pro-inflammatory transcription factors and cytokines. Alternatively, phosphorylation of the GR by p38 MAPK has also been demonstrated to potentially contribute to GCR [47].

Hair cortisol concentration is emerging as a reliable biomarker of chronic stress and has been linked to discrimination over time [48–50]. Urinary cortisol has also been used as a biomarker and found to associate with lower SES in the Multi-Ethnic Study of Atherosclerosis (MESA) [13]. Additionally, glucocorticoid-related gene expression has been analyzed, particularly when examining the impact of intergenerational trauma on the HPA axis [51]. In animals, the HPA axis and immune response to social stress has been investigated. In mice, HPA activation and corticosterone production during repeated social defeat stress release monocytes into circulation and induce glucocorticoid resistance of myeloid cells [52]. Research in rhesus macaques demonstrate that social status may affect glucocorticoid-mediated gene expression [53]. Similar social factors have also been reported to affect the HPA axis in humans. The stress of social evaluation has specifically been shown to increase the cortisol response [54]; lower positive social support has also been shown to increase the cortisol response [55]. Such investigations provide more supporting evidence for the potential effects of SDoH on the HPA axis and provide mechanisms whereby SDoH may lead to poorer health outcomes. More research is needed to comprehensively understand the signaling pathways mediating the effects of SDoH on diseases like CVD.

The effects of chronic stress on CVD in particular have been well documented [56–58]. Similarly, there have been numerous reports of cortisol associating with CVD and CVD risk factors. In a prospective cohort study performed at a tertiary care center with 3052 participants who underwent coronary angiography, increased morning serum cortisol was associated with adverse cardiovascular risk factors including elevated systolic blood pressure, higher fasting glucose, increased triglycerides, and lower glomerular filtration rate [59]. In this study, increased cortisol also associated with higher counts of CD16+CD56+ (NK cells) and CD3+CD8+ (T-suppressor) cells. The author notes these are all only cross-sectional associations, though their evidence is in line with various additional investigations [60–63], including results from a 2019 Jackson Heart Study [64]. In a case–control study which included 500 angiographically confirmed coronary atherosclerosis patients and 500 age and sex-matched controls having no coronary stenosis, elevated hair cortisol proved to be the most significant predictor of coronary atherosclerosis in a multivariable analysis of CVD risk factors [65]. These results are in line with numerous other investigations [66,67], including reports of elevated hair cortisol preceding acute myocardial infarction (AMI) [68,69], and associations between hair cortisol and a history of CVD [70].

#### Corticotrophin-releasing factor (CRF)

CRF, also known as corticotropin-releasing hormone (CRH), is released by the hypothalamus in response to stress and orchestrates the activation of the HPA axis as described above. CRF released due to chronic stress has been investigated for its influence on emotions such as fear and anxiety, as well as negative homeostatic disturbances on many organ systems including cardiovascular, gastrointestinal, immune, and reproductive systems [71,72]. Among the many effects of CRF, perhaps the most widely investigated clinical implication has been the role of CRF in mental illness [73]. Hyperactivity of the HPA axis has been implicated as one of the main drivers of psychiatric disorders [74], and increased levels of CRF in the CSF of suicide victims and patients experiencing depression have been historically reported [75–79]. Furthermore, environmental influences on genetically susceptible individuals may also play a role as certain SNPs in the CRFR1 gene appear to mediate the development of depression following trauma in childhood [80,81]. In mice, chronic social stress induced longstanding demethylation of the CRF gene [82,83], and transgenic mice over-expressing CRF in the brain exhibited signs of depression and anxiety [83–85].

CRF and related peptides known as urocortin 1–3 bind to two types of receptors called corticotropin-releasing factor receptor 1 (CRF1R) and 2 (CRF2R) [40]. Biological activity of these 4 peptides is regulated by the CRF-binding protein (CRFBP) [86]. Interestingly, CRF1R and CRF2R are expressed not only in the brain but throughout the nervous system and peripheral tissues, including immune cells, which underscore the broad biological effects of CRF and related peptides in behavioral, cognitive, neuroendocrine, immune, and autonomic responses to stress [87–90]. CRF1R and CRF2R are class B GPCRs, mainly mediated by stimulatory G protein (Gs) coupling [90,91]. Activation of CRF receptors results in Gabg-receptor signaling ultimately leading to activation of PKA- PKC, cAMP, and ERK-dependent signaling pathways initiating AP1, CREB and NF-kB activation [89]. CRF1R is more widely expressed in the brain than CRF2R while CRF2R has higher expression levels in peripheral tissues, particularly the heart [92], and plays a role in blood pressure [93,94]. CRFR-mediated cAMP signaling has been shown lead to phosphorylation of several proteins involved in the excitation of cardiomyocytes including L-type Ca2+ channels, phospholamban, ryanodine receptor (RyR), and troponin 1 [93]. CRF has been shown to increase cardiac function through activation of PKA, CaMKII and AKT signaling pathways, [95] though chronic activation may lead to detrimental effects [93,96,97]. With chronic activation, CRFR desensitization has been reported to be initiated by phosphorylation through GRKs, PKA, or PKC, and involves β-arrestin recruitment and receptor endocytosis with clathrin-coated vesicles [73].

Given the intersection of SDoH, chronic stress, mental health [98–105], and dysregulated CRF signaling, it is imperative that future studies exploring SDoH of mental illness incorporate analyses of CRF and downstream signaling pathways both centrally and peripherally. Such investigations may provide a mechanistic understanding for how SDoH associate with mental health outcomes.

#### Adrenocorticotropic hormone (ACTH)

ACTH signals through the melanocortin receptor 2 (MC2R) ultimately increasing intracellular cAMP levels and activating PKA-ERK1/2 downstream signaling including CREB-mediated regulation of transcription [106]. ACTH secretion has been found to be altered in obstructive sleep apnea and depression [107], and has been investigated for use as a biomarker of chronic stress [108].

### Other biomarkers of stress

In addition to dopamine, epinephrine, norepinephrine, cortisol, CRF, and ACTH, chronic stress also induces a variety of other mediators, neurotransmitters, and neuropeptides which regulate an organism’s ability to respond to stressors [108].

#### Serotonin

Serotonin, a neurotransmitter important for mood regulation and widely utilized target of selective serotonin reuptake inhibitors in depression treatment, has been shown to be dysregulated in chronic stress and associate with depressive symptoms [109]. Synthesized from L-tryptophan, an essential amino acid, serotonin signals through serotonin receptors of which more than 15 so far have been identified. Serotonin activates several key signaling pathways including PI3K/AKT, MAPK, PKC, as well as cAMP/PKA which subsequently initiates CREB- and NF-κB-dependent pathways [110]. The neurochemical mechanisms of serotonin's association with chronic stress have been explored in mice exposed to chronic unpredictable stress; adult male rats exhibited cognitive dysfunction similar to that of depression as well as selective cell death in the interfascicular nucleus of the dorsal raphe, which may impair serotonergic innervation of the medial prefrontal cortex. As such, serotonin has been explored as a biomarker of chronic stress [111].

Serotonin can also be found outside of the CNS and has different functions in the periphery, such as enhancing the interaction of platelets with tissue factor-rich microvesicles, promoting procoagulant activity [112]. Serotonin is secreted by enterochromaffin cells of the digestive tract and stored in platelets. This circulating stock may be captured by sympathetic neurons and endothelial cells [113]. It has been theorized that circulating serotonin may play a role in systemic arterial hypertension [114] and high circulating serotonin has been reported to be a risk factor for coronary artery disease, particularly in younger age groups [115]. Moreover, 5-HT_4_ receptors have been detected in normal adult human heart tissue and may play a role in the progression of heart failure [116]. It should also be noted that serotonin seems to both directly and indirectly modulate NK cell function [117]. Whether in relation to depression, chronic stress, the immune system, or CVD, serotonin may be a useful factor to investigate in future studies of the biological effects of SDoH.

#### Oxytocin

The neuropeptide oxytocin has been connected to major depressive disorder (MDD), post-traumatic stress disorder (PTSD), has been shown to increase during acute stress, and is known to regulate social connection, social behavior and emotions [118]. Additionally, in patients with MDD, it has been shown that higher oxytocin levels drive the association between increased social support and decreased perception of loneliness [119]. When oxytocin binds to its receptor, several signaling pathways activate including PKC, p38 MAPK, and ERK1/2 with subsequent activation of CREB- and MEF-2 mediated *de novo* protein synthesis. Interestingly, oxytocin has been postulated to play a protective role in the cardiovascular system [120], highlighting the importance of further research aiming to understand oxytocin’s role in the connection between chronic stress and CVD, and how its potential protective function could be altered by chronic stress or traumatic experiences related to SDoH.

#### Brain-derived neurotropic factor (BDNF)

BDNF, a neurotrophic factor, is derived in the hippocampus and downregulated by glucocorticoid levels as well as serotonergic projections during the acute and chronic stress response [121], which is at least partially influenced by DNA methylation of the BDNF gene [122]. BDNF signals through the tropomyosin-related kinase B (TrkB) subsequently activating ERK1/2 and PI3K pathways [123]. Interestingly, BDNF has been postulated to be up-regulated by exercise and exhibit cardioprotective functions [124,125].

#### Dehydroepiandrosterone (DHEA)

DHEA is a steroid hormone secreted from the adrenal glands known to reduce pro-inflammatory responses and alter lipid metabolism. DHEA concentrations are tightly connected to HPA axis activity and increased in acute and chronic stress [126,127].

Several biomarkers of stress have been linked to various SDoH and chronic stress. Interestingly, the activated signaling pathways downstream of these stress biomarkers seem to include overlapping key signaling molecules such as p38 MAPK, ERK1/2, CREB, and NF-κB. Future research should focus on these shared pathways when examining SDoH and CVD.

### The role of inflammation in the biology of adversity

The chronic exposure to adverse SDoH have ultimately been linked to an increase in inflammation accompanied by an imbalance of pro- versus anti-inflammatory cytokines.

#### Glucocorticoid Resistance (GCR) in inflammation

As outlined above, several biomarkers of stress, especially cortisol, are canonically anti-inflammatory and blunt stress-induced inflammation. However, a seemingly paradoxical increase in inflammation is associated with chronically elevated cortisol levels over time which can, in part, be explained by GCR as discussed above [22]. GCR occurs when immune cells respond less strongly to cortisol after becoming desensitized following decreased surface expression of GR. This reduced GR translocation to the nucleus prevents the inhibition of NF-kB-mediated transcription. Thus, GCR results in enhanced *de novo* protein synthesis of pro-inflammatory cytokines such as TNFα and IL-6 [128]. Acquired GCR has been linked to chronic PSES including increased frequency of discrimination, decreased social support, increased social isolation, and depression [22,129–134]. Furthermore, blunted diurnal cortisol slopes have been associated with poor health and CVD outcomes, and supporting evidence was recently compiled in a systematic review and meta-analysis [135].

Unfortunately, only a few psychosocial factors or SDoH have been systematically analyzed regarding their role in acquired GCR. Future translational studies [136] should include PBMC-related GCR in characterization of the stress response, as direct measurement of stress biomarkers may not wholly explain the impact of chronic PSES on CV risk and CVD. While various mechanisms leading to glucocorticoid receptor dysfunction have been described [137], it is important to understand the connection between GCR and health outcomes in the context of PSES related to SDoH. Proposed biological mechanisms include epigenetic changes like methylation of the glucocorticoid receptor gene itself, or possibly involvement of the proteasome [138,139].

#### Dopamine signaling and inflammation

DA is less well studied than cortisol or the other catecholamines in the setting of SDoH and chronic PSES, but has been implicated in pro-inflammatory and immune-cell modulating signaling pathways [140]. For example, it has been demonstrated that D1 signaling promotes NRLP3 inflammasome ubiquitination and autophagy-mediated degradation resulting in inhibition of IL-1b production [141]. These findings are in accordance with other evidence demonstrating that signaling from DA receptors with less affinity to DA (i.e., D1 and D2) relates to anti-inflammatory events, while higher affinity receptors like D3 and D5 might be involved in pro-inflammatory action in a potentially dose-dependent manner [140]. In activated CD4+ T cells, D3 signaling has been found to increase IFNγ secretion and decrease IL-4 and IL-10 production. Furthermore, stimulation of D3 significantly decreases intracellular cAMP levels and TCR-induced ERK2 phosphorylation, contributing to the differentiation of CD4+ T-cells into the inflammatory Th1 phenotype. D5 signaling in LPS-stimulated dendritic cells selectively increase IL-12 and IL-23 production and induce differentiation into the inflammatory Th17 phenotype [142,143]. There is a need to further understand the importance of DA and its receptors in CV risk and CVD, especially given that an agonist to the DRD2 was used in a Phase 1 clinical trial for Parkinson’s disease and has been associated with cardiovascular complications like orthostatic hypotension and heart failure [144]. DA receptors have also been connected with diabetes development, where enhanced DA levels due to chronic PSES could potentially be a contributing factor [145]. However, translational studies linking DA receptor signaling with inflammation or immune cell dysfunction in the setting of chronic PSES and CVD are rare.

#### Inflammatory cytokines and chemokines in the biology of adversity

Individuals residing in disinvested and disadvantaged neighborhoods most impacted by structural discrimination with increased crime, violence, decreased walkability, low levels of social cohesion, decreased access to healthcare, increased pollutant exposure, and food insecurity display higher serum levels of CRP, IL-6, and fibrinogen [146]. Nazmi et al. also reported that higher levels of safety are associated with decreased IL-6 levels even after adjustment for race, ethnicity, and SES [146]. In a recent study from our own laboratory, we demonstrated that within a cohort of African Americans, neighborhood socioeconomic deprivation as measured by a neighborhood deprivation index (NDI) was associated with increased levels of TNFα and IL-1β [147]. We also determined that neighborhood deprivation was positively associated with TMAO [147], a biomarker associated with the microbiome, CV risk factors, and CVD mortality [148,149]. Interestingly, our data show that the NDI-to-TMAO relationship was significantly mediated by the TNFα and IL-1β [147], indicating the overall significant role of systemic low-grade inflammation in potentially accelerating CVD through multiple mechanisms, including effects related to the microbiome [150].

When comparing lower-SES to higher-SES individuals as determined by income or educational attainment, CRP was higher in the disadvantaged group [151]. In the Framingham Offspring study, Loucks et al. discovered that, in the fully adjusted model, SES when determined by educational level remained significantly associated with CRP, sICAM-1, and MCP-1. All are known biomarkers connected to cardiovascular risk and CVD itself [152]. A meta-analysis of 43 published papers (*N* = 111,156) found that lower SES is associated with increased CRP and IL-6 [153]. Interestingly, in a study comparing the impact of an acute stressor in men with lower versus higher SES, men from the lower SES group displayed a prolonged IL-6 increase and heart rate increase when compared with the higher SES group. While this particular study could not find baseline differences by SES, it clearly demonstrates an altered inflammatory response that directly affects the cardiovascular system upon exposure to an acute stressor [154].

An increase in circulating IL-6 levels has especially been seen in aging adults residing in neighborhoods with racial segregation and increased poverty [155]. In a study with 380 clinically healthy adults, high levels of perceived stress predicted various plasma cytokine levels including IL-6, TNFα, MIP-1α, and MIP-1β [156]. Numerous different stressors have been connected to increased inflammation including early childhood stressors [157], racial discrimination, poverty, neighborhood disadvantage, war, displacement, and famine [158,159]. Risky emotional family environment in childhood, for example, predicted higher levels of IL-2, IL-6, IFNγ, and TNFα in adulthood [160]. In another study, exposure to lower SES in early life resulted in increased CRP levels in adulthood when compared with individuals growing up with higher SES [161]. We have recently summarized, in the context of COVID-19, various other individual-level adversities affecting inflammation, especially IL-6 [158]. This includes lack of social cohesion, experience of physical assault in women, loneliness, sleep deprivation, bullying, sexual assault, high levels of ‘anticipatory racism’ threat, perceived lifetime discrimination, as well as ongoing discrimination. It is also important to recognize that many other factors associated with these SDoH, like limited healthcare access, but unrelated to the stress response may contribute to adverse health effects, and more research is needed to examine these intersectional and complex determinants of health.

Discrimination and inflammation appear to be connected independent of age according to various studies [159,162–165], with potentially a greater impact in women [166]. In a multi-ethnic study, associations between lifetime discrimination and inflammation burden (as determined by a composite score of various pro-inflammatory cytokines) were significant even in models that controlled for age, gender, education, medication use, smoking, alcohol use, depression, and anxiety). Furthermore, poor sleep quality [167] mediated the association between lifetime discrimination and inflammation.

Asberg and colleagues in 2009 began investigating differences in various cytokine levels among women healthcare employees in Sweden who scored high on a questionnaire assessing professional burnout when compared with healthy women control workers [168]. Increased levels of vascular endothelial and epidermal growth factor (VEGF and EGF) were found in women experiencing high levels of chronic stress due to burnout, indicating their potential use as stress biomarkers. Interestingly, a 2009 follow-up study by Jonsdottir et al. attempted to replicate these results in a Swedish population of women patients diagnosed with Exhaustion Disorder, a disorder characterized by chronic stress-induced exhaustion related to stressors experienced over at least a period of 6 months. In contrast with previous results, Jonsdottir and colleagues failed to find significant associations with any cytokines measured except for C-reactive protein (CRP) [169]. The authors concluded VEGF and EGF might not be valuable biomarkers of stress especially when compared across multiple studies due to differences between analytical techniques, storage time, differing study populations, and large inter-individual variation. Despite these concerns, more recent studies have found success using VEGF and EGF as biomarkers of stress. In 2016, Wallensten and colleagues again examined cytokines associated with Exhaustion Disorder among women patients in Sweden and found significantly higher levels of circulating VEGF and EGF compared with controls [170]. Additionally, an intervention study found EGF may be a potential biomarker for anxiety and depression, given that cognitive behavioral therapy and mindfulness were found to significantly decrease EGF levels [171].

These multiple studies from Sweden examining VEGF and EGF in populations of women experiencing chronic stress and burnout are especially relevant to Black women in the United States, a population at a higher risk of experiencing burnout in their daily life and career [172,173].VEGF [174] and EGF [175] have both been connected with atherogenesis and CVD in mice and in humans, further indicating the importance of examining these biomarkers in relation to chronic stress and CVD outcomes.

The association between inflammation and social inequality have been the subject of recent reviews and meta-analyses, both in humans and across species [22,153,176–178]. However, longitudinal studies are needed to determine the potential effects of behavioral interventions related to dietary changes or physical activity on cytokine levels and CV risk. Additionally, translational studies with appropriate animal models might be needed to further understand the biological pathways involved to (1) better understand if and why therapies investigated in clinical trials might not be as successful in individuals experiencing underlying chronic PSES and (2) identify targets for interventions to reduce the effects of adverse social conditions. We have recently highlighted this interdisciplinary approach in a commentary published in Cell [179].

### Immune cell distribution and function in the biology of adversity

#### Neural–hematopoietic axis

Psychosocial and environmental stress may impact proliferation and migration of immune cells.

**Figure 2:**
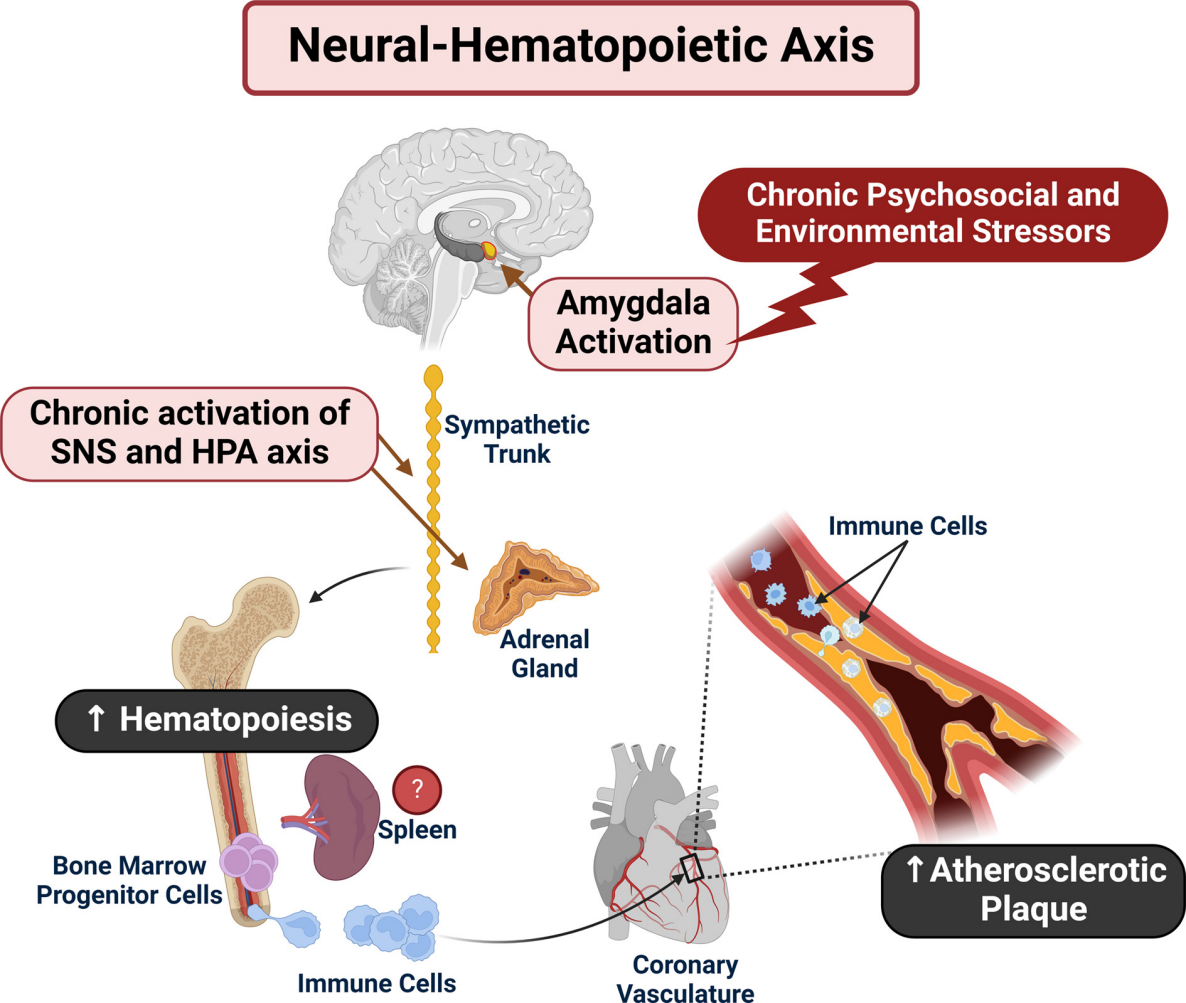
The neural–hematopoietic axis and chronic PSES Using 18F-FDG PET/CT, chronic PSES have been shown to increase activity of the amygdala which has been associated with increased activity of the bone marrow and spleen as well as arterial inflammation. It is hypothesized that sympathetic afferents stimulate hematopoiesis in the bone marrow, and possibly spleen, which results in increased migration of immune cells to arteries and may lead to cardiovascular disease.

Our lab and others have found that amygdala activity, as measured by ^18^Fluorodeoxyglucose Positron Emission Tomography Computed Tomographic (^18^FDG-PET/CT), associates with chronic stress, CVD, and increased bone marrow activity or hematopoiesis [6,7]. In murine models of chronic stress, noradrenergic SNS afferents were discovered in the bone marrow which may be one pathway by which chronic stress-associated neural activity may activate hematopoiesis [180]. This pathway is collectively called the neural–hematopoietic axis and may explain the migration of inflammatory immune cells to atherosclerotic plaques (Figure 2) [7,181,182]. While metabolic activity of bone marrow was elevated in these investigations, our study also found elevated activity in the spleen which has been reported to act as a secondary site of hematopoiesis during periods of stress [183,184].

We must also consider the plausibility of stem cell proliferation in the bone marrow and its potential relationship to clonal hematopoiesis, which has recently been connected to inflammation and CVD [185]. Findings like these should certainly be explored in individuals experiencing chronic PSES, as activation of the sympathetic nervous system has been implicated in hematopoietic stem cell proliferation in mice [186]. It will be crucial to determine if stress-related hematopoiesis could also be driven by chronic activation of the SNS in humans [187].

#### Immune cell characterization

**Figure 3:**
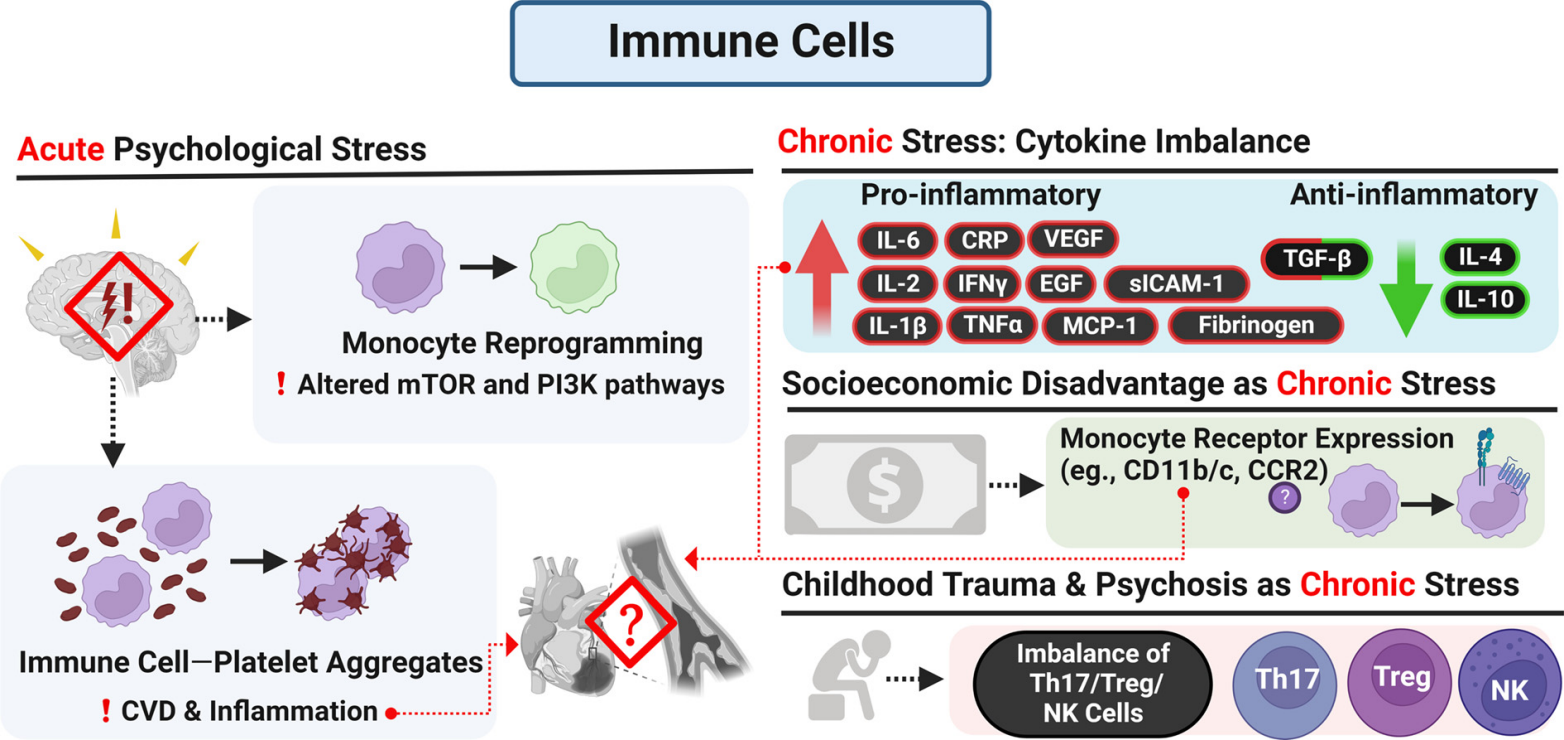
The impact of chronic PSES on the immune system PSES have been reported to alter immune cell distribution, immune cell function, and induce chronic inflammation partially due to an imbalance of pro-/anti-inflammatory cytokine levels. Effects of both acute and chronic stress have been reported, and each has been connected to CVD. Acute stress has been shown to alter monocytes as well as increase immune-cell platelet aggregates, which may be related to atherosclerosis. Chronic stress due to PSES has been shown to shift cytokine expression towards pro-inflammatory signaling. Specific chronic PSES are highlighted, such as socioeconomic disadvantage which has been associated with altered monocyte receptor expression. Childhood trauma and psychosis as chronic stressors have been linked to an imbalance of Th17, Tregs, and NK cells which may affect immune function.

Given that chronic stress may induce hematopoiesis and potentially contribute to an altered immune response, it is important to determine if PSES associate with specific immune cell profiles or cellular subsets found in circulation (Figure 3). While there is evidence from macaque and mouse studies that chronic stressors like social status alter the immune cell landscape [177,188], larger human cohort studies examining immune cell profile and CV risk are rare [189,190]. For example, in a study amongst acute coronary syndrome (ACS) patients wherein the mean percentage of monocytes, lymphocytes, and neutrophils relative to each other were 6.9%, 25.4%, and 64.7%, it was found that after controlling for LV function and hospital arrival time, adverse life-events and hostility associated with increasing levels of monocyte frequency, while emotional support associated with a decrease in monocyte frequency [191], potentially indicating that adverse SDoH might impact recruitment of monocytic cells to atherosclerotic plaques and further accelerate unstable plaque features. These findings highlight the need for further research on how SDoH alter monocytes beyond distribution by assessing receptor expression profiles and function. Meaningful associations were also found for % neutrophils [191]. In a study of healthy female volunteers, the level of monocytes in circulation was increased by acute stress, independent of the monocyte subtype [192]. Students experiencing acute exam stress were found to display reduced absolute numbers of monocytes and NK cells, while NK cells as well as T cells displayed shifts in their subtype profiling [193]. One psychological variable examined in this study was ego depletion which refers to a state of impaired self-control and is associated with increased perceptions of fatigue or lack of energy and motivation/willpower. Interestingly within this student group, students with a higher level of ego-depletion displayed lower absolute NK cell numbers. The authors hypothesize these results may reflect a suppression of NK cell-immunity as a physiological response to preserve energy, induced by the perceived mental fatigue exhibited by increased ego depletion [193]. In women with eating disorders, NK cell redistribution was significantly altered after encountering an acute psychosocial stressor. However, when women engaged in coping strategies, the acute stress response of NK cells was blunted [194]. Reduced and altered stress response in NK cells has also been studied in the setting of psychosis and psychosis with childhood trauma [195]; changes in T cells were also reported. In patients with a high psychosis liability (high risk of psychosis or recent-onset psychosis), decreased NK cell numbers and increased T regulatory cells predicted increased subjective stress in response to social stressors. In patients with both high psychosis liability and a history of childhood trauma, increased Th17 cells were observed. Similarly, a study in teachers found that overcommitment and work-life imbalance were connected to lower NK cell numbers; additionally, an overall decrease in T-helper cell numbers was observed [196].

In terms of acute psychological stress, studies have found associations with platelet activity as determined by immune cell – platelet aggregates, which are associated with CVD risk [197–200]. A recent study in mice and humans undergoing stressful events showed monocytes were reprogrammed towards an activated or hyperresponsive inflammatory state by altering mTOR and PI3K pathways [201]. This reprogramming was accompanied by chromatin alterations in gene regulatory regions at inflammatory loci, further supporting a growing list of genes impacted by chronic and acute stress.

Immune cell receptor expression is a second important step of immune cell characterization that should be considered after examination of cellular subsets. SES and low CNS-serotonin function were shown to increase the expression of CD11b/c on monocytes and by this, potentially promote atherogenesis [202]. In a study on acute stress in females, acute stress was found to upregulate CSF-1 receptor and CD29, while CD62L displayed trends towards increased expression [192]. When examining NK cells of students undergoing acute exam stress, increased CD62L expression was accompanied by decreased CD57 and KLRG1 expression, indicating a shift toward immature NK cells [193]. Similarly, reduced CD57-positive NK cell proportions were found in individuals experiencing greater life stress [203].

Determination of immune cell function can act as a third step in characterizing the effects of PSES on immune cells and subsequent CVD risk, especially in relation to immune cell-derived cytokine production. A major assay to analyze the function of immune cells in the setting of chronic stress is the detection of glucocorticoid resistance (GCR). For this assay, lipopolysaccharide (LPS) or another stimulator is used to induce a pro-inflammatory state in immune cells; glucocorticoid is then added at increasing concentrations [204]. The mean concentration of glucocorticoid needed to blunt LPS-induced cytokine production by 50% (e.g., dexamethasone IC50) is used as the primary measure of GCR. This assay has been utilized to examine the negative impact of racial discrimination on immune cells [131].

Several studies have also determined the ability of immune cells to produce cytokines without incorporating GCR assays. When immune cells of community volunteers were stimulated with LPS and the secretion of various cytokines were measured, IL-8 release was found to be associated with depression and perceived stress [205]. However, increasing levels of perceived social support were associated with decreasing IL-8 release by LPS-stimulated immune cells [205]. In a study examining immune cell function in students experiencing acute exam stress, increased IL-6 and TNFα secretion was found after LPS stimulation [193]. A study in teachers examining the impact of work-life balance found that, while at baseline IL-6 was increased and IL-10 decreased, the release of TNFα upon leukocyte stimulation was accelerated in teachers with a higher work–life imbalance. Additionally, the secretion of IL-2 was increased in individuals with higher overcommitment [196]. Psychological stress may also affect the expression of anti-inflammatory cytokines, potentially reducing their expression leading to increased inflammation. Chronic stress was found to decrease levels of IL-10 in rat models, and IL-10 as well as IL-4 levels were decreased in medical students who exhibited high anxiety the day before an examination [206,207]. Cytokines with both anti- and pro-inflammatory functions such as transforming growth factor β (TGF-β) exhibit mixed results in the setting of chronic stress [207,208].

While the analysis of cytokines provides a window into the functioning of immune cells, it can also be useful to examine gene expression more broadly with DNA microarrays or high throughput technologies like RNA sequencing. Using these methods, chronic stress or adversity was found to associate with altered expression of a specific group of genes termed the Conserved Transcriptional Response to Adversity (CTRA) [209]. CTRA encompasses a set of 53 genes regulated by β-adrenergic signaling, glucocorticoid signaling, as well as pro-inflammatory stimuli [161]. Pro-inflammatory CTRA genes were shown to be up-regulated in African Americans with high scores of perceived racial and ethnic discrimination. Interestingly, controlling for perceived discrimination scores in multivariable analysis significantly attenuated race-related differences in CTRA expression, highlighting the independent role of discrimination in CTRA [210]. Furthermore, early life adverse social conditions in macaques recapitulate the characteristic CTRA expression pattern, with transcript origin analysis showing the alterations were embedded within the basal transcriptome of CD4+ T lymphocytes and B lymphocytes [211].

### Methylation and biology of adversity

**Figure 4:**
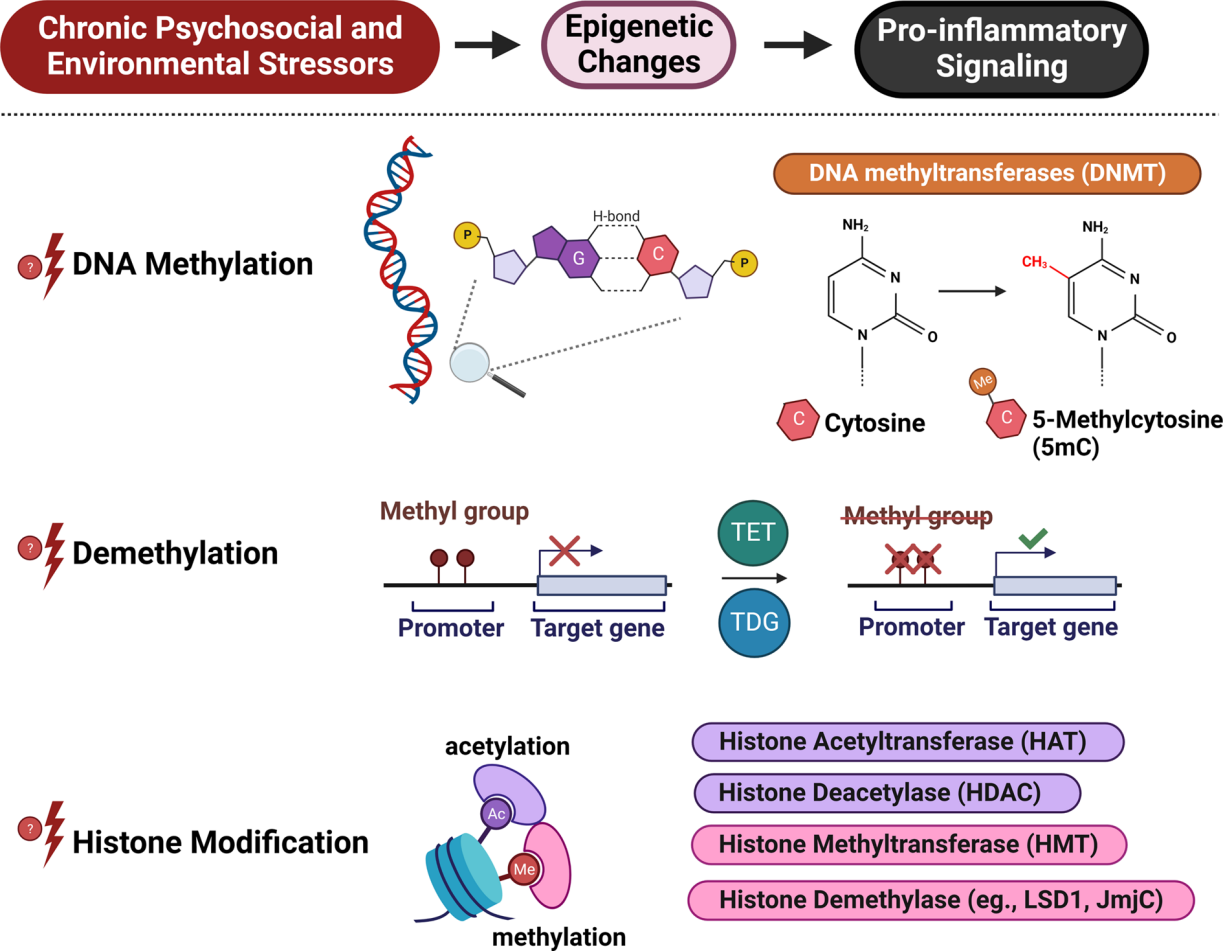
Chronic PSES induce epigenetic changes which promote proinflammatory signaling Various PSES have been shown to lead to DNA methylation and demethylation as well as histone modification, all of which affect expression of genes. The addition of a methyl group to cytosine serves to block transcription of the affected gene, while the removal a methyl group can promote transcription. Histones can be modified by the addition or removal of acetyl or methyl groups which also affect transcription. Through an interplay of these mechanisms, PSES have been shown to alter cytokine and immune cell responses potentially accelerating atherogenesis.

Epigenetics refers to molecular mechanisms that modify the expression of genes without altering their underlying DNA sequences by addition or removal of methyl or acetyl groups from genetic chromatin structures (Figure 4). The process of demethylation is accomplished by the ten eleven translocation (TET) and thymine DNA glycosylase (TDG) enzymes, while deoxyribonucleic acid methyltransferase (DNMT) methylates the 5-position cytosine of a gene’s chromatin structure. Additionally, histone acetyltransferase (HAT) acetylates lysine on the histone proteins of a gene’s chromatin structure, while the process of deacetylation is regulated by histone deacetylase (HDAC). More research in epigenetics is needed to better understand how gene expression modifications occur in response to adverse social conditions [212]. As described in a recent study which found associations between SDoH and epigenetic changes among childhood cancer survivors [213], previous research has demonstrated epigenetic changes specifically with SES [214–219], education [220,221], and cigarette smoking [222].

#### Evidence for epigenetic regulation of stress response

In response to acute stressors, glucocorticoid production is regulated by end-production inhibition, which generates a negative feedback response targeting the hypothalamus and anterior pituitary gland [223]. Translocated glucocorticoid receptors are influenced by epigenetic mechanisms that modify enzymatic functioning through mechanisms involving DNA methylation [224], histone methylation [225], and histone acetylation [226], potentially leading to GCR. The neuron specific glucocorticoid receptor gene NR3C1 has been reported to be impacted by epigenetic changes after childhood abuse [227]. Additionally, maternal depression during pregnancy has been linked with increased fetal methylation of glucocorticoid receptor (GR) genes, which in turn were linked with greater cortisol stress responses in infants at 3 months post-birth [228]. In rats, maternal care practices such as licking, grooming, and arched back nursing resulted in decreased methylation of hippocampal GR genes, as well as altered histone acetylation which persisted into adulthood and associated with decreased stress responses [229]. Additionally, mouse models demonstrate that parental abuse results in increased methylation of BDNF genes that both persist into adulthood and are seen in the offspring of the maltreated mice [230]. Given that BDNF deficiency has been linked to learning disabilities [231] and other neurocognitive disorders, the impact of parental abuse on BDNF methylation points to an important way by which epigenetics mediates relationships between social experience and adverse health outcomes.

Similarly, β-adrenergic receptors have been shown to be impacted by epigenetic modifications. Methylation of the *ADRB2* gene have been associated with decreased asthma symptoms in inner city children [232]. As β-adrenergic receptor density has been described to be impacted by SDoH [24], a potential regulation by epigenetic mechanisms is indeed feasible. Notably, PSES hypomethylate *ALOX15*, *CAPN14*, and *POSTN* asthma genes which increases the prevalence of asthma symptoms [233]. The prevalence of this relationship was found to be three times greater in Americans of African descent than Americans of European descent [234]. Researchers have also noted the upregulation of histone acetylation at the D2-like receptor in the medial prefrontal cortex and amygdala in the setting of anxiety [235]. Additionally, it is important to note that early life stressors were found to induce MAO-B DNA methylation, potentially leading to dysfunction of monoaminergic receptor signaling, which provides one plausible mechanistic link between SDoH and mental disorders as well as potential pitfalls in future treatment responses [237].

One important way that social determinants impact epigenetics is through trauma and its associated stress response, especially in early life. In individuals who have experienced severe childhood abuse, researchers identified several hundred sites of consistent hypermethylation or hypomethylation compared to controls [238]. Similar trends were observed in DNA methylation of oligodendrocyte genes in individuals who had committed suicide when comparing those with or without a history of child abuse [239]. Additional studies are needed to establish the signaling pathways connecting these experiences of trauma and the resulting observed epigenetic changes. Nevertheless, these associations emphasize the relationship between lived experience and epigenetic changes.

#### The biology of adversity – telomere length and acceleration of the ‘iAge clock’

**Figure 5:**
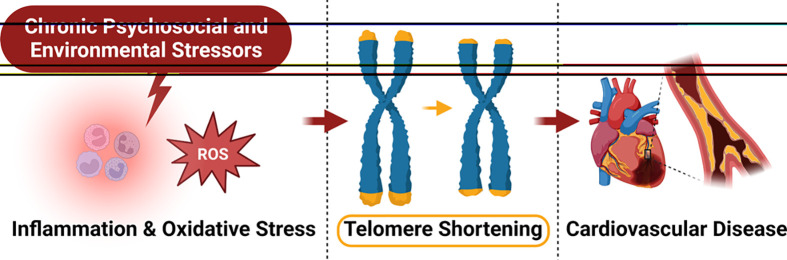
Chronic PSES induce telomere shortening Chronic PSES have been shown to shorten telomeres potentially via increased inflammation and oxidative stress. Shortened telomeres have been associated with CVD.

Telomere length has been studied extensively in relation to SDoH [240], is related to a variety of diseases including CVD [241], and has been shown to highly associate with DNA methylation age [242] (Figure 5). Interestingly, it has been demonstrated that young Black individuals display greater telomere length when compared with White individuals; however, this apparent advantage is lost over the lifetime due to an accelerated rate in telomere shortening [243]. Inflammation and oxidative stress are the main mechanisms reported to advance the loss of telomere length; both mechanisms, detailed in this review, are ultimately connected to chronic PSES [244–247]. The list of PSES that affect telomere length and/or telomerase activity is long but worth mentioning, as many unfortunately experience these stressors on a daily basis: neighborhood disadvantage [248,249], neighborhood deprivation [250,251], low SES [252,253], early life stress [254], lower social support, lower optimism, increased early life adversity, high hostility [255], anxiety [256], and racial discrimination together with internalized negative racial bias [256–258].

Over the past decade, scientists have developed several epigenetic aging scores and at least two of those (DNAmPhenoAge and Grim Age) are associated with increased CVD risk [259]. Calculation of epigenetic age uses DNA methylation data incorporated into complex algorithms determining one’s ‘biological age’ [260,261]. Very recently, Schmitz et al. determined in the MESA study that SES significantly associated with GrimAge and the DunedinPoAm clock, which seemed independent of health behaviors or factors like smoking, alcohol consumption, or obesity. Thus, the authors concluded that social disadvantage might lead to accelerated biological age [262]. Evans et al. recently highlighted key studies incorporating age clock measures and includes detailed measures of SDoH in diverse populations [263].

## Limitations

In this review article, we aimed to provide an overview of mechanisms by which adverse SDoH might impact the biology of the human body aiming to focus on signaling pathways which in the past have been connected to the experience of PSES. This way, we demonstrate that the stress response is a possible signaling pathway through which SDoH gets under the skin to influence human biology. However, we recognize that various SDoH domains or social exposures are not interchangeable; hence, marginalized individuals and minoritized persons do not experience the negative effects of SDoH in isolation, rather, they exist at the intersection of multiple vulnerabilities [264] and have to be considered separately or in conjunction with each. Therefore, we recommend that investigators and clinicians employ a critical intersectional lens in their research and clinical practice respectively, particularly when examining the complex role of SDoH in CVD development and management. This approach will aid in disentangling the complex SDoH phenomenon to better understand the intersectional effects of its domains on CVD disparities. Additionally, employing an intersectional approach to highlight the lived experience (e.g., everyday experience of discrimination, racism, and violence) of marginalized populations, to be connected to the complex changes on the cellular level, will personify the ‘biology of adversity’ and the role of individual SDoH domains in CVD development and progression, aiming to better develop targeted behavioral intervention to reduce CVD risk in the most vulnerable of our society. Finally, we acknowledge that the review is reflective of the most current literature and cannot summarize all of the related studies. Our expectation is that this review will initiate dialogue and research ideas aimed at extricating the complex pathways underlying the impact of SDoH on CVD risk to facilitate the development of future interventions to enhance equity.

## Conclusion

**Figure 6:**
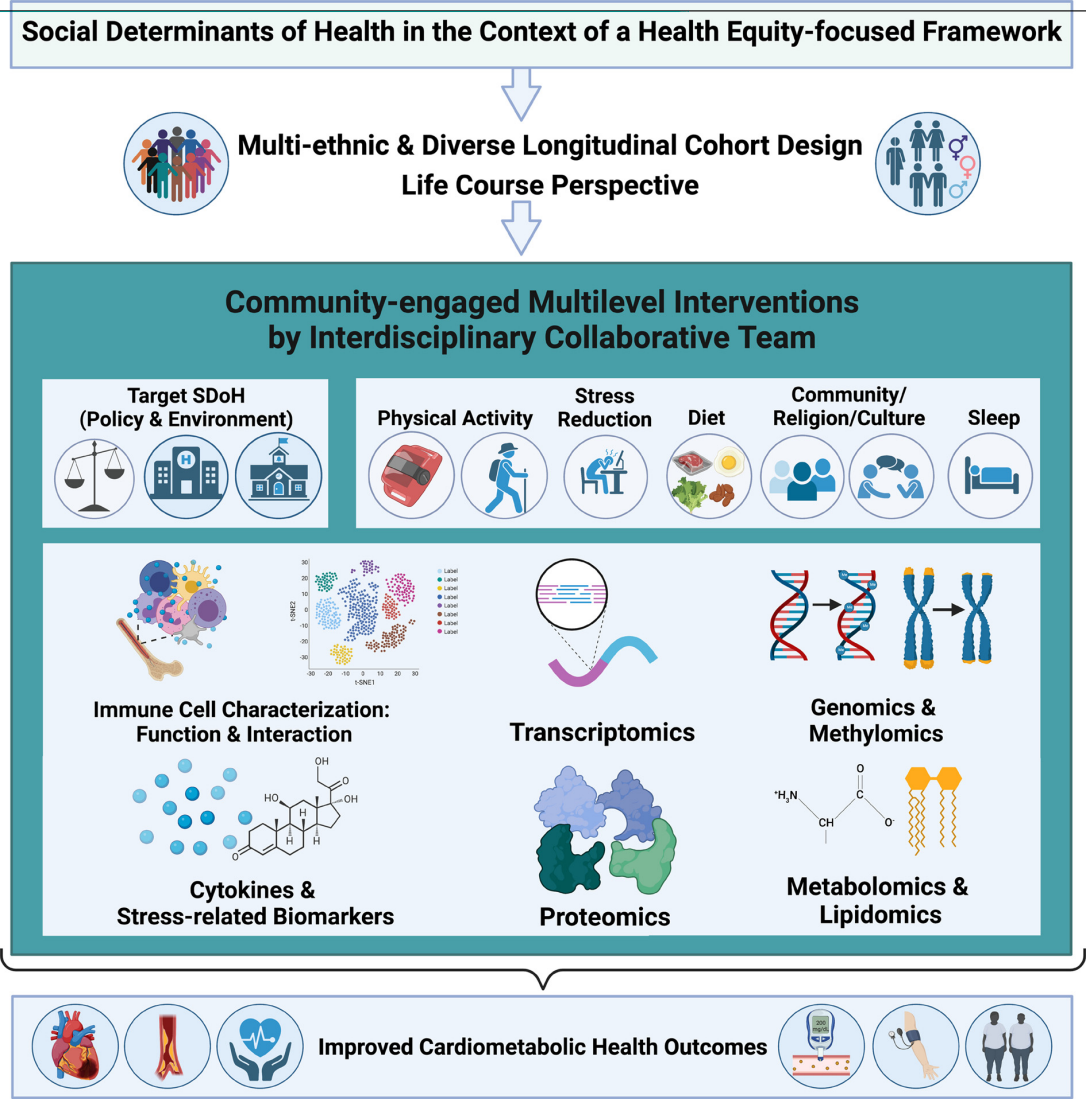
Future research directions In this review, we have highlighted studies which have investigated the impact of chronic stress or stress-related biomarkers on cellular signaling pathways. However, there are several important directions for future research. (1) Future research should be performed by interdisciplinary teams of epidemiologists, basic/translational, behavioral, and social scientists, clinicians, and statisticians with active engagement of the community in which the research is being done. (2) Interdisciplinary teams need to work with communities who have been historically underrepresented and marginalized in biomedical research using established community engagement methods as many referenced studies in this review inadequately represent diverse populations including Asian, Latinx, non-Hispanic Black, Native American/Alaska Native, and Native Hawaiian/Pacific Islander populations as well as sexual and gender minority populations of all racial/ethnic groups. (3) Teams should use longitudinal studies over the life course to examine signaling pathways and markers (i.e. immune cell characterization, various ‘-omics’, and cytokines and stress-related biomarkers) as mediators of the relationships between social determinants of health (SDoH) and cardiometabolic health factors. (4) Teams should also create multi-level interventions which simultaneously target both policies that impact SDoH (i.e. housing, education, occupation, physical and social neighborhood environment) and behaviors related to cardiometabolic health (i.e., physical activity, stress, reduction, diet, and sleep). (5) These multi-level interventions can be used to investigate key signaling pathways linked to cardiometabolic outcomes and most amenable to health behavior change.

More work is needed to fully elucidate the biological pathways through which social inequalities lead to health inequalities. As a whole, the work presented here demonstrates key avenues for future research and substantiates the overall importance of investigating the molecular mechanisms governing the biology of adversity and chronic stress (Figure 6).

While this review focuses on the negative effects of SDoH, we should also highlight studies that have demonstrated protective SDoH such as hope, social and family support [130,265–267], compassion [160], and strong racial identity [268] which may at least partially overcome the impact of PSES on inflammation, telomere length, and/or epigenetic aging [269]. Future studies should combine epidemiologic research with a strong basic/translational research component to further elucidate connections between SDoH and biological pathways [270]. Such research will be broad in scope by design. Future studies can further develop and investigate emerging SDoH, such as markers of neighborhood social environment based on real-time global positioning system (GPS) data, as more proximal markers of environmental exposures. Future work can also investigate the connections between neuroscience and the immunology of the stress response, to understand more detailed mechanisms that connect the brain to atherosclerotic plaque development in at-risk, minoritized, and/or disadvantaged populations. With many potential avenues of research, each piece important and interdependent, we strongly emphasize the need for extensive collaboration between experts in epidemiology, psychology, neuroscience, immunology, vascular biology, and public health with funding mechanisms that allow for this type of interdisciplinary work. The emerging field of artificial intelligence may also play a key role, especially given the many interrelated factors governing SDoH and CVD. The incorporation of machine learning to analyze the multiple layers of various omics -approaches, cytokine, and immune cell measurements, as well as clinical parameters with SDoH could help to establish patterns and networks to better understand the complexity of the biology of adversity.

It is also important to consider SDoH and CVD processes over the life course. Research focusing on early CVD pathogenesis may need to consider different social determinants or signaling mechanisms than research focusing on later CVD progression, or CVD among different populations. For example, structural and intermediary SDoH, most prominently perceived stress and perceived social support, have been found to associate with preterm delivery [271]. Preterm delivery has been shown to associate with an increased risk of CVD for both the mother [272], and the child later in life [273]. Certainly, the biological mechanisms governing CVD risk, development, and progression may differ among these two populations throughout the life course despite influences from the same SDoH. Similarly, SES has been shown to affect HF patient outcomes, in part due to disparities in access to care [274]. This mechanism of SES affecting HF patient outcomes may have separate biological mechanisms than those associated with developing HF among a population of low SES who experiences chronic stress from living in an unsafe, resource-limited community [275]. Attention to the life course and context of SDoH is paramount to disentangling their many effects on CVD.

Better understanding the biology of adversity is crucial to design multi-level interventions that reduce CVD disparities and promote health equity. At the individual level, this understanding can inform individualized patient care that assesses and addresses SDoH across the life course [1,276]. Community- and policy-level interventions are also needed to address the upstream structural factors, such as healthcare access, and SDoH and their impact on cardiometabolic health across populations [1]. Ultimately, these interventions should be informed by the community and must be available, accessible, and affordable to persons of low SES to equitably reduce the impact of SDoH and the biology of adversity in CVD disparities [1] (Figure 6).

## Supplementary Material

Supplementary Figure S1

## Data Availability

This is a review article and no original data hence available.

## Abbreviations

ACS: acute coronary syndrome
ACTH: adrenocorticotropic hormone
AR: adrenergic receptor
BDNF: brain-derived neurotropic factor
cAMP: cyclic adenosine monophosphate
CNS: central nervous system
CREB: cAMP response element binding protein
CRF: corticotrophin-releasing factor
CRF1R: corticotrophin-releasing factor receptor 1
CRF2R: corticotrophin-releasing factor receptor 2
CRFBP: corticotrophin-releasing factor binding protein
CRH: corticotropin-releasing hormone
CRP: C-reactive protein
CTRA: Conserved Transcriptional Response to Adversity
CV: cardiovascular
CVD: cardiovascular disease
D1LR: dopamine 1-like receptor
DA: dopamine
DARPP-32: 32-kDA dopamine and cAMP-regulated phosphoprotein
DHEA: dehydroepiandrosterone
DNMT: deoxyribonucleic acid methyltransferase
EGF: epidermal growth factor
Epi: epinephrine (adrenaline)
ERK: extracellular signal-regulated kinase
GCR: glucocorticoid resistance
GPCR: G-protein-coupled receptor
GR: glucocorticoid receptor
GRE: glucocorticoid response element
HAT: histone acetyltransferase
HDAC: histone deacetylase
HF: Heart Failure
HPA: hypothalamic–pituitary–adrenal
iAge: inflammatory aging clock
IFNγ: interferon γ
IL: interleukin
LPS: lipopolysaccharide
LTSS: long-term separation stress
MAPK: mitogen-activated protein kinase
MCP-1: monocyte chemoattractant protein 1
MDD: major depressive disorder
MESA: Multi-Ethnic Study of Atherosclerosis
MKP-1: mitogen-activated protein kinase phosphatase-1
NDI: neighborhood deprivation index
NE: norepinephrine (noradrenaline)
NF-κB: nuclear factor κB
NK cells: natural killer cells
PBMC: peripheral blood mononuclear cell
PKA/C: protein kinas A/C
POMC: Pro-opiomelanocortin
PSES: psychosocial and environmental stressors
PTSD: post-traumatic stress disorder
RyR: Ryanodine receptor
SAM: sympatho-adrenomedullary
SDoH: Social Determinants of Health
SES: socioeconomic status
SGK1: serum/glucocorticoid regulated kinase
SNS: sympathetic nervous system
STSS: short-term separation stress
TDG: thymine DNA glycosylase
TET: ten eleven translocation
TGF-β: transforming growth factor β
TNFα: tumor necrosis factor α
TrkB: tropomyosin-related kinase B
VEGF: vascular endothelial growth factor
