# Supplementary material for: By what molecular mechanisms do social determinants impact cardiometabolic risk?
Source: Clin Sci (Lond). 2023 Mar 24;137(6):469–94. doi: 10.1042/CS20220304 (PMC10039705; doi:10.1042/CS20220304)
Supplement: Supplementary Figure S1 [file CS-2022-0304C_supp.pdf]

## Supplementary Figure

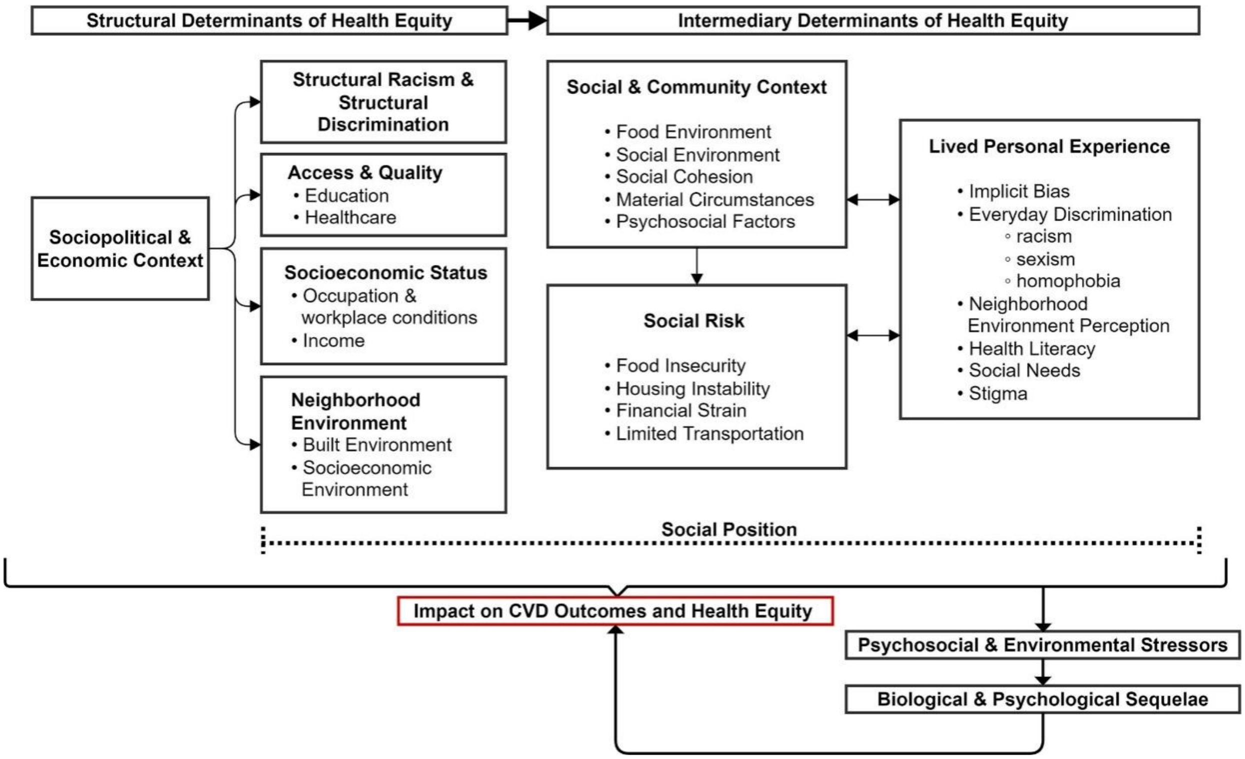


**Figure S1. Social determinants of health framework described by Powell-Wiley et al.** (1).This innovative framework of Social Determinants of Health (SDoH)combines structural determinants such as socioeconomic status (SES) and neighborhood environment, with intermediary determinants including the social environment and lived personal experiences of discrimination. SDoH have been reported to serve as sources of chronic psychosocial and environmental stress (PSES) and are associated with a variety of diseases like cardiovascular disease.

## Supplementary References

1. Powell-Wiley TM, Baumer Y, Baah FO, Baez AS, Farmer N, Mahlobo CT, et al. Social Determinants of Cardiovascular Disease. Circulation Research. 2022;130(5):782-99.
